# Prevalence of psychoactive substance use among youth in Rwanda

**DOI:** 10.1186/s13104-015-1148-2

**Published:** 2015-05-08

**Authors:** Maurice Kanyoni, Darius Gishoma, Vedaste Ndahindwa

**Affiliations:** Physiotherapy Department, School of Health Sciences, College of Medicine and Health Sciences, University of Rwanda, Kigali, Rwanda; Mental Health Department, School of Nursing and Midwifery, College of Medicine and Health Sciences, University of Rwanda, Kigali, Rwanda; Department of Epidemiology and Biostatistics, School of Public Health, College of Medicine and Health Sciences, University of Rwanda, Kigali, Rwanda

**Keywords:** Substance use, Dependence, Youth, Prevalence, Rwanda, Nicotine, Alcohol, Cannabis

## Abstract

**Background:**

Substance use among youth is a significant public health concern worldwide. However, little is known in Rwanda about the prevalence of drug use among youth. The goal of the current study was to assess the prevalence and determinants of substance use among youth in Rwanda.

**Methods:**

A cross-sectional home survey was carried out with 2479 Rwandan youth. Youth ranging, in age from 14–35 years, were randomly selected from 20 out of the 30 districts in the country. The youth were interviewed using a questionnaire that included socio-demographic information and self-reported substance use. Misuse and dependence on alcohol, marijuana and tobacco were respectively assessed by the Alcohol Use Disorders Identification Test (AUDIT), the Cannabis Abuse Screening Test (CAST), and the Hooked on Nicotine Checklist (HONC).

**Results:**

Overall, the prevalence rate of substance use over the month prior to the survey was 34% for alcohol, 8.5% for tobacco smoking, 2.7% for cannabis, 0.2% for glue and 0.1% for drugs such as diazepam. 7.46% (one in thirteen) of the youth were alcohol dependent, 4.88% (one in twenty) were nicotine dependent, and 2.54% (one in forty) dependent on cannabis.

**Conclusions:**

Our findings demonstrate that tobacco, alcohol, marijuana and other substance use are realities in the daily lives of youth in Rwanda. Further research is needed to monitor the evolution of this phenomenon and its determinants and in order to initiate evidenced-based interventions.

## Background

The use of alcohol, tobacco, cannabis and other psychoactive substances constitutes one of most important public health problems among youth worldwide [1]. Recent studies in African countries have shown that the phenomenon of drug use is also common in this continent and is becoming one of the most disturbing health-related problems among youth [2]. Studies show that there is an increasing incidence in the use, and a decreasing age of onset, of these substances [3,4]. Most young people begin their use of drugs with alcohol and cigarettes and later progress to more dangerous substances such as cannabis and cocaine [5].

Little is known in Rwanda however about the prevalence of drug use among youth and hence the gravity of the problem is not known. Based upon frequent field work observations and reports from police and hospitals, in 2011 the Ministry of Youth in collaboration with the Kigali Health Institute, sponsored a nationwide research project to explore the prevalence of substance use among adolescents and young adults in Rwanda. Therefore, the current study was designed to provide baseline data to guide prevention strategies and to inform youth organizations, government, and the Ministries of Health, Education and Youth. This study is important because it was conducted in Rwanda, a country in the process of rebuilding after the genocide. The genocide destroyed family and community protection factors for thousands of young people.

## Methods

### Research design

To establish the prevalence and identify risk factors associated with substance use among adolescents and young adults in Rwanda, we conducted a community household-based study with a cross-sectional, descriptive design from June to November 2011.

### Participants

The target population for this study was youth between 14 and 35 years. This age range was chosen because in Rwanda the Ministry of Youth defines youth as “everyone in the age bracket of 14 to 35 years” [6]. The designation of “youth” in Rwanda includes two groups of individuals, adolescents and young adults. Based on the frame for the Rwanda Population and Housing Census (RPHC) provided by the National Institute of Statistics of Rwanda and using the Multistage sampling design a sample of 2479 youth (adolescents and young adults) was recruited. Rwanda is divided into 5 provinces and each province is sub-divided into districts, sectors, cells and villages. In Rwanda there are 30 districts, 417 sectors and 14,837 villages. The average village size is 610 residents, made up of 133 households. The total population in Rwanda is 10,412,826, with youth representing almost 37% of the population and those under 14 representing 42% (The Republic of Rwanda, NISR, [7]; The Republic of Rwanda, NISR, [8]).

In the first stage of recruitment, two thirds of all districts in Rwanda were randomly selected. The probability of selection for each district was proportional to the number of districts in each of five provinces in Rwanda. Firstly, we have used 2/3 due to resources constraints. Secondly, sampled districts had similar characteristics to other districts not included. Using this methodology, 20 out of 30 districts were selected to participate. During the second stage of sampling, using the probability proportional to size sampling, 4 administrative sectors in each district were selected (4*20 = 80 sectors) and in each sector, one village was selected. For the final sampling stage, we used a complete list of households with names of all residents and using a systematic sampling frame, 28 households per village were selected. Therefore, the total number of households visited was 2240 and all youth aged between 14- 35 living in selected households were systematically recruited. In some households there were no youth however this was compensated for by including youth from households with more than one youth. Selected households without any youth in the range of 14–35 years old represented approximately the ratio of 1:4. Households with more than one youth were found were also approximately in the ratio of 1:4 and interviewed youth in that case per household were 2 or 3.

In total, 2479 youth were interviewed, and of these 56.0% were males and 44.0% females. As indicated previously, the age range of the participants was 14 to 35 years of age with a mean of 23.2 years, a median of 23 years and a standard deviation (SD = 5.52). The mean number of youth recruited in each district was 124 youth (range 89-150). As is common throughout Rwanda, our respondents were more likely to live in rural than urban areas (83.3% vs 16.7%). 45.8% of youth had both parents alive, 39.7% had one parent alive while 14.4% of the youth reported that both parents were dead. Most participants (60.0%) reported their marital status as single, about one-third (37.6%) were married and the remaining (2.4%) were widowed, divorced or separated. 23.2% of the youth were students, 12.0% had completed school and 51.1% have dropped out of school. Almost two thirds (61.4 %) of participants were living in families designated as poor, 35.1% lived in resourceful families and 3.4% live in rich families (the money rich, the food rich) using the “Ubudehe classification”^a^.

### Measures

- A structured questionnaire with thirty-one close-ended questions was used. This questionnaire was specifically designed to obtain socio-demographic information from participants, including information about the use of alcohol, tobacco, cannabis and other drugs. The lifetime use of a substance was defined as ‘ever using any of the substances in a lifetime (e.g. ‘Have you ever smoked any of following substances?’), while recent use was defined as the use of any of the substances in the last 12 months. Continuing use was defined as use within the 30 days preceding the survey.
- Participants who reported using alcohol, tobacco, or cannabis were additionally asked about the magnitude (abuse and dependence) of consumption. Substance abuse and dependence data were obtained using 3 instruments (AUDIT, CAST and HONC – see below).
- The Alcohol Use Disorders Identification Test (AUDIT): to date, more than 25 questionnaires screening for alcohol dependence have been described in the literature and many studies have shown that their sensitivity is at least equal to laboratory tests [9]. The AUDIT questionnaire is a simple and widely used tool developed by the World Health Organization for the early identification of misuse and dependence on alcohol. This tool is a 10-item screening questionnaire with 3 subscales: the first scale includes 3 questions on the amount and frequency of drinking; the second scale includes 3 questions on alcohol dependence; and the third scale includes 4 questions on problems caused by alcohol [10]. The original tool was developed in English and was translated in Kinyarwanda by a professional translator. Finally the tool was back translated to English by a different professional translator. The authors compared the two versions of the tool and found them to be equivalent. The questionnaire was pre-tested among youth from 4 *imidugudu* (villages_ in Kigali that were not villages sampled for our study. The internal consistency for the consensual Kinyarwanda version of the questionnaire was found to be acceptable (Cronbach’s alpha = .860).
- The Cannabis Abuse Screening Test (CAST) is a 6-item scale designed for adolescents and young adults to identify problematic forms of cannabis use that might lead to negative social and health consequences. The major advantage of this instrument is that it is brief and easy to administer [11,12]. Similarly to the AUDIT tool, the CAST was translated into Kinyarwanda and back-translated back to English. The internal consistency for the Kinyarwanda version of the tool was also acceptable (Cronbach’s alpha = .808).
- The Hooked on Nicotine Checklist (HONC) is a 10-item instrument used to determine the strength of tobacco dependence. It is a reliable and valid measure of diminished autonomy over tobacco and it is used with smokers of all ages [13]. The number of positive responses in the instrument reflects the degree of dependence. The internal consistency of the Kinyarwanda version (following back translation) was acceptable (Cronbach’s alpha = .893).

Previously trained research assistants were given house-hold lists with the houses to be sampled highlighted in yellow to show the approximate location of the household on the sketch map. The questionnaire was administered during a home visit by research assistants (RAs). The RAs had a background in social, psychology and medical sciences and were trained in the use of the survey instruments.

### Ethical considerations

The current study was approved by the Ethical Review Board of Kigali Health Institute (KHI), the National Institute of Statistics of Rwanda (NISR) and the Ministry of Youth Republic of Rwanda (MINIYOUTH).Verbal permission to interview the eligible youth was also sought and granted from the local authorities (from the sector, the cell and the village responsible). We explained to the youth that participation was voluntary and for those who participated their identification would remain anonymous and confidential. The objectives and significance of the research were explained to the respondents and consent forms signed. Participants who could not write were asked to have a witness sign on their behalf. The informed consent of minors (aged below 18 years in the Rwandan context) was obtained from the respective parents or guardians. During the data collection, names were replaced by codes to maximize anonymity. Participants who reported alcohol or drug abuse were referred to centres and hospitals near their homes for follow-up care. Raw data will be kept for 5 years in locked cupboards in the research unit of Kigali Health Institute (KHI).

### Statistical analysis

Primary data was coded and entered using Epi Info version 3.1 while prevalence rates for substance use, abuse and dependence were computed using then STATA 11 software. Chi-square analyses were used to compare these rates and socio-demographic variables.

## Results

### Prevalence rates of substance use among youth in Rwanda

#### Lifetime prevalence (experimentation)

In the present study, youth were asked if they had ever used one or more psychoactive substances. The results (Table 1) demonstrated that more than half (52.5%) of the participants reported that they had used one or more substances at least once in their life time. Thus, the overall lifetime prevalence rate for substance use among youth in Rwanda (n = 2479) was 52.5% (50.6%-54.5%, 95% Conf. Interval). The mean age of onset (of all substances under study) has been found to be 11.4 year (Me = 11years, SD = 6.43). Generally the youth tried more than one substance: 50.6% of the youth had used alcohol; 10.6% tobacco; 4.4% cannabis; .5% solvents; and 0.1% had used drugs such as diazepam. In addition 1% had used local brews with a mixture of sorghum, sugar and cannabis.

**Table 1:** **Lifetime prevalence of substance use among youth in Rwanda**

	**Have ever used any substance**		**p**
	**Yes (%)**	**No (%)**	
**Gender**			
Male	67.03	32.97	0.000
Female	36.92	63.08	
**Residence area**			
Rural	55.61	44.39	0.000
Urban	45.12	54.88	
**Status of parents (Alive)**			
Yes, both	50.22	49.78	0.000
Yes, one	54.97	45.03	
No	62.61	37.39	
**Age groups**			
10-15	30.77	69.23	0.000
16-20	39.95	60.05	
21-25	59.03	40.97	
26-30	65.55	34.45	
31-35	68.54	31.46	
**Student status**			
Still a student	32.63	67.37	0.000
Finished studies	57.29	42.71	
Dropped out of school	59.12	40.88	
Never went to school	66.96	33.04	

The results demonstrated that the proportion of male youth using substances (67.03%) was nearly double that of females (36.92%); this difference was statistically significant (p < 0.001). Youth from rural areas were more likely to experiment with substances than those from urban areas (p < 0.001). This was quite a surprising result because we had expected to have more users in urban areas. Status of parents and student status was also been found to have an influence on drug use. Being a student was associated with low rates of substance experimentation while dropping out of school and never going to school were associated with a high prevalence rate of substance use.

Youth without parents were more likely to use drugs than those with one or both parents (p < 0.001). We also found that many youth who used alcohol, tobacco and marijuana came from families where other family members used drugs (p < 0.001). The family members included parents, spouse, brothers and sisters and other members of the extended family staying with them. Table 1 demonstrates that the rate of substance use increases as youth get older (p < 0.001). However, we did not found any significant difference in usage rates relative to current marital status, level of education of the head of household, and the socio economic category of youth households (*p >* 0.15).

#### Past 30 day and 12 months prevalence

Lifetime prevalence of use (whether the person has ever used the drug) is a good measure of youth experimentation. Past 12 months prevalence and past-30 day prevalence (whether the youth has used the drug within last year or the last month) are good measures of recent and current use (European Monitoring Center for Drugs and Drug Addiction, [14,15]). During the 30 days and 12 months prior to participation, alcohol was, by far, the most widely reported substance used, at 34% and 39% respectively. The next most commonly used was cigarettes: 9.5% of youth reported using cigarettes in past 12months cigarettes and 8.5% had used cigarettes in the past 30 days. Only a small proportion of youth (0.2%) reported using glue in the past 12 months and the same proportion had used glue in the previous 30 days. Although rates tended to decrease from the lifetime prevalence (experimentation) to the current and continuing prevalence of drug use, the past-30-day prevalence rates demonstrate that substance use is currently a reality in the daily lives of youth in Rwanda. A promising finding was that youth who had used one or more substance during the previous 30 days or 12 months were not necessarily addicted to the drug. To examine this particular phenomenon, we used additional indicators to track the level of addiction to substances.

### Dependence

#### Alcohol

We used the Alcohol Use Disorders Identification Test (AUDIT) developed by the World Health Organization for the early identification of misuse and dependence, with our sample of alcohol users (n = 967), and found (Table 2) that 493 (50.98%) of this group had a total score in the range of 0-7. This score indicate normal use or absence of alcohol related problems. 289 (29.88%) of our sample achieved an AUDIT score in the range of 8-15 representing a medium level of alcohol dependence. Scores of 16 and above represent a high level of alcohol dependence. We found that among those individuals who drank alcohol during previous 12 months (n =967), nearly 20% of them (n = 185) were alcohol dependant. It is this last group that WHO recommends being considered as having a drinking problem. In other words, 7.46% of our total sample (185/2479) were alcohol dependent based on the WHO tool. Dependence and misuse on alcohol was found to be significantly associated with gender, age, having parents alive and being a student (p < 0.001). However rates of alcohol dependence did not differ significantly based on where the youth lived, his/her marital status, the level of education by the head of household and the socio economic category of youth households (*p >* 0.300).

**Table 2:** **Twelve months, past-30 day prevalence and dependence among youth in Rwanda**

	**Lifetime prevalence (experimentation)**	**Past 12 months Prevalence (Recent use)**	**Past-30-day prevalence rates (continuing use)**	**Dependence**
**Alcohol**	50.6%	39%	34%	7.46% (based on AUDIT score)
**Cigarettes**	10.6%	9,5%	8,5%	4.88% (based on HONC score)
**Cannabis**	4.4%	2.9%	2.7%	2.54% (Based on CAST score)
**Glue**	0.5%	0.2%	0.2%	Not assessed
**Drugs such as Diazepam**	0.1%	0.1%	0.1%	Not assessed

AUDIT: The Alcohol Use Disorders Identification Test; HONC: The Hooked on Nicotine Checklist; CAST: The Cannabis Abuse Screening Test.

#### Cannabis

The misuse of cannabis was assessed using the Cannabis Abuse Screening Test (CAST). The CAST assessed problematic use of and dependence on cannabis. The results show that 10.18% of youth had a mild risk of becoming dependant while 58.33% were already dependant. Dependence and misuse on cannabis was also found to be significantly associated with gender, age, residential setting and having parents alive (Table 3). However misuse and dependence were not significantly associated with being a student, current marital status, the level of education of the head of household and the socio economic category of youth households (p > 0.133).

**Table 3:** **Alcohol ad Cannabis dependance and sociodemographic profile**

	**Alcohol misuse**			**P**	**Cannabis Misuse**			***P***
	**Social users (%)**	**On risk (%)**	**Dependence (%)**		**Social users (%)**	**On risk (%)**	**Dependence (%)**	
**Sex**								
Male	68.33	18.66	13.02	0.000	94.65	0.8	4.56	0.000
Female	96.69	2.85	0.46		100	0	0	
**Residence area**								
Rural	80.86	11.95	7.19	0.343	97.72	0.29	1.99	0.000
Urban	80.63	10.41	8.96		93.46	1.21	5.33	
**Status of parents (alive)**								
Yes, both	83.36	9.56	7.08	0.000	98.14	0.09	1.77	0.009
Yes, one	80.82	12.14	7.04		96.33	0.82	2.86	
No	72.75	17.13	10.11		95.22	0.56	4.21	
**Age groups**								
10-15	96.15	3.85	0	0.000	100	0	0	0.032
16-20	90.18	7.12	2.7		98.04	0.37	1.6	
21-25	77.19	13.16	9.65		96.2	0.88	2.92	
26-30	73.33	15.74	10.93		96.67	0	3.33	
31-35	71.05	16.45	12.5		95.39	0.66	3.95	
**Student status**								
Still a student	94.58	4.37	1.05	0.000	98.25	0.35	1.4	0.133
Finished studies	77.1	13.13	9.76		97.31	0	2.69	
Dropped out of school	76.21	14.2	9.6		96.59	0.4	3.01	
Never went to school	78.53	13.24	8.24		96.18	1.18	2.65	

#### Tobacco

The misuse of cigarettes was assessed using the Hooked on Nicotine Checklist (HONC). Results demonstrated (Table 4) that 29.66% of the youth were at risk of becoming dependant while 46% were already dependant on nicotine. On this basis, we can conclude that 4.88% of our total sample (121/2479) has problems of dependence on nicotine. As for alcohol and tobacco, all of the demographic factors were statistically significant (p < 0.001) with the exception of residential setting, current marital status, level of education of the head of household and the socio economic category of youth households (*p >* 0.300).

**Table 4:** **Tobacco dependance and sociodemographic profile**

	**Tobacco misuse**			***P***
	**Social users (%)**	**On risk (%)**	**Dependence(%)**	
**Sex**				
Male	85.47	6.07	8.46	0.000
Female	99.26	0.37	0.37	
**Residence area**				
Rural	92.08	3.50	4.42	0.039
Urban	88.62	4.12	7.26	
**Status of parents (alive)**				
Yes, both	94.60	1.77	3.63	0.000
Yes, one	89.69	4.69	5.61	
No	86.52	6.46	7.02	
**Age groups**				
10-15	99.23	0.77	0.00	0.000
16-20	96.69	1.72	1.60	
21-25	90.94	4.24	4.82	
26-30	86.30	4.81	8.89	
31-35	84.87	6.25	8.88	
**Student status**				
Still a student	97.38	1.4	1.22	0.000
Finished studies	91.58	5.39	3.03	
Dropped out of school	89.77	3.73	6.5	
Never went to school	87.94	5.29	6.76	

## Discussion

Tobacco, alcohol, marijuana and other drug use are prevalent in Rwandan youth. The current research provided preliminary data on the prevalence of substance use among youth in Rwanda. The findings from the present study revealed that the past-30 day prevalence among youth was 34% for alcohol, 8.5% for tobacco smoking, 2.7% for cannabis, 0.2% for glue and 0.1% for drugs such as diazepam. The mean age of onset of drug use was 11.4 years. Other similar research studies have shown that adolescent and young adult period are particular moments of vulnerability because it is tempting to experiment with drugs. This finding is significant in terms of the development of health related behaviours because adolescence is a time when many new behaviours are explored, some of which may become established and continue through to adulthood [16-18]. Beyond the stage of experimentation or usage, some youth were found to be misusing or dependent on alcohol. The study revealed that 7.46% of the youth were alcohol dependent, 4.88% were dependent on nicotine and 2.54% were dependent on cannabis. Various variables associated with drug use, including age, gender, residential area, being student, having parents alive have been presented.

Several studies have been published on the prevalence of drinking, smoking and other illicit drug use among youth but it is difficult to compare rates between countries, given the differences of context, culture and methodology. Variations in instruments used, age group considered, and population characteristics such as residential status, make comparisons difficult. In addition, information on drug use in Africa is still extremely limited, given the lack of scientific surveys in the region [19]. Despite those limitations, the available research indicates that some studies have used similar questions to investigate the lifetime and past 30 day prevalence of drug use. These findings provide some insights into the significance of our results. The prevalence rates of drug use among Rwandan adolescents and young adults fall with-in a range similar to other African countries. The prevalence of alcohol use in Kenya is quite high with a lifetime prevalence of 57.9% for respondents aged 14-23 years [20] and a current alcohol prevalence of 50.7% % for respondents aged 18-32 years [21]. A lower prevalence rate (9.2%, N = 402) of alcohol use has been reported among Nigerian youth between 11 and 20 years in one study [1] however another study [22] reported a lifetime prevalence of 66% for alcohol use among Nigerian youth. In South Africa, Reddy et al. [23] reported that 49.1% of adolescents between 13 and 19 years had consumed one or more drinks of alcohol in their lifetime and 31.8% had consumed one or more drinks in the previous month. In Ghana, lifetime prevalence of alcohol use varied from 25.1% (N = 894) among youth aged 18 to 24 years [24] to 39.3%, (N = 1195) among youth between 12 and 18 years. [25].

Cannabis is also commonly used among young people in Africa. In South Africa 12.8% of youth reported ever using cannabis and 9.1% had used cannabis on one or more days in the past month [23]. The lifetime prevalence for cannabis use was found to be 18.3% (N = 458) among youth aged 14-23 years in Kenya [20], 4.4% (N = 402) among Nigerian youth aged 11-20 years [1] and 2.6% (N = 894) among Ghanaian youth 18-24 years [24].

Tobacco use by young people is also of major concern in Africa. According to Reddy et al. [23] 30.5% of South African youth 13-19 years had smoked cigarettes in their lifetime while 21.1% acknowledged consuming tobacco during the past 30 days. The lifetime prevalence for tobacco use among youth aged between 11 and 20 years was reported to be 20% in Nigeria [22], 34.7% in Kenya [20], and 7.5% in Ghana [24].

Though the prevalence in Rwanda is relatively low compared to other countries in the region this might be explained by the fact that most if not all of its neighbours have access to big commercial trade from Asia and South American Countries which are thought to be origins of the used drugs. The increasing rate of substance use in Rwanda however might be due to genocide and breakdown in family ties.

## Conclusion

Results from this study highlight the issue of substance use among adolescents and young adults in Rwanda. Youth, parents, teachers, civil society and public institutions concerned with the prevention and treatment of drug related-problems must be informed of the prevalence of drug use among Rwandan youth. We recommend that early intervention target students and out-of-school youth in urban and rural areas in order to prevent drug use at early age.

## Endnote

^a^The Ubudehe socio-economic classification is a tool used in Rwanda to evaluate the income/resource of household based on local standards. The local administration meets collectively with the population in each village of Rwanda in order designate the category each household fall in. The Ubudehe socio-economic classification is comprised of six categories described by Kettlewell [26]: (1) *Umutindi nyakujya* (those in extreme poverty): is someone who needs to beg to survive. They have no land or livestock and lack shelter, adequate clothing and food. No access to medical care; children are malnourished and do not attend school. (2) *Umutindi* (the very poor): Same as 1 but physically capable of working on land owned by others, very small land holdings, no livestock. (3) *Umukene* (the poor): Have some land and housing. Live on their own labour and produce, and though they have no savings, they can eat, even if the food is not very nutritious. (4) *Umukene wifashije* (the resourceful poor): same as 3 but may have small ruminants and their children go to primary school. (5) *Umukungu* (the food rich): larger land holdings on fertile soil and enough to eat. Own livestock, often have paid jobs, and can access health care. (6). *Umukire* (the money rich): Have land and livestock, and often have salaried jobs. Good housing, often owns a vehicle, and has enough money to lend and to get credit from the bank.
